# Ameliorative effect of aqueous avocado seed extract against chromium‐induced oxidative stress and cellular damage in rabbit kidney

**DOI:** 10.1002/fsn3.4210

**Published:** 2024-05-22

**Authors:** Hanan A. Okail, Sadia Anjum, Nahed M. Emam, Rewaida Abdel‐Gaber, Mohamed A. Dkhil, Saeed El‐Ashram, Mona A. Ibrahim

**Affiliations:** ^1^ Department of Zoology, Faculty of Science Sohag University Sohag Egypt; ^2^ Biology Department, Faculty of Science Hail University Hail Saudi Arabia; ^3^ Department of Zoology, Faculty of Science Al‐Arish University Arish Egypt; ^4^ Department of Zoology, College of Science King Saud University Riyadh Saudi Arabia; ^5^ Department of Zoology, Faculty of Science Helwan University Cairo Egypt; ^6^ Applied Science Research Center Applied Science Private University Amman Jordan; ^7^ College of Life Science and Engineering Foshan University Foshan Guangdong Province China

**Keywords:** avocado, chromium, histopathology, kidney, oxidative stress

## Abstract

The accumulation of chromium in renal tissues promotes the generation of reactive oxygen species (ROS), leading to oxidative stress, genomic and cellular harm, and ultimately necrotic and apoptotic cell death induced by free radicals. Hence, the utilization of antioxidant phytochemicals becomes crucial for cellular defense against oxidative damage. This study endeavors to explore the potential protective effects of an aqueous avocado seed extract (ASE) on rabbit kidneys exposed to chromium‐induced damage. Fifteen adult rabbits were distributed into three groups: Group 1 was kept as the control. The second and third groups received a daily dose of K_2_Cr_2_O_7_ (5 mg/kg) intraperitoneally for 2 weeks. While the third group was given an oral dose of ASE (400 mg/kg). In rabbits administered with Cr (VI), kidney homogenates showed a marked increase in Malondialdehyde (MDA) (69.3 ± 4.1 nmol/g) along with a decrease in glutathione (59 ± 5.8 nmol/mg) content and the activity superoxide dismutase (SOD) (0.5 ± 0.05 U/mg protein), glutathione peroxidase (GPx) (16.7 ± 1.1 μmol/mg protein), and catalase (CAT) (73.8 ± 3.9 U/g protein) compared to the levels in control group. Also, the gene expression data for the enzymes SOD, GPx, and CAT dropped dramatically in kidney tissue following Cr (VI) injection. Additionally, Bowman's capsule and glomerulus showed degenerative alterations in the kidney's histopathology and immunohistochemistry. ASE treatment when administered along with Cr (VI) enhanced the activity and gene expression of antioxidant enzymes and improved histopathological conditions. The findings of this study unequivocally show that avocado seed extract, which is rich in phenolic derivatives, is a potent nephroprotective agent that inhibits nephrotoxicity induced by Cr (VI) in rabbits.

## Graphical abstract

Chromium accumulation triggers ROS production, causing oxidative stress and cell damage. ASE, an antioxidant phytochemical, defends cells against chromium‐induced oxidative damage. ASE treatment in rabbits enhances antioxidant enzyme activity, improves histopathological conditions, and shows potent nephroprotective effects.

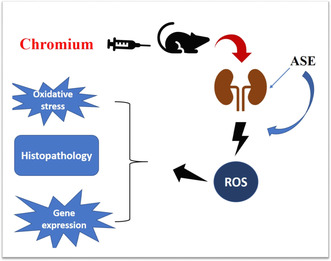

## 1 INTRODUCTION

Chromium (Cr), identified as the most toxic heavy metal ion (Gupta, 2015), naturally exists in the earth's crust in a variety of oxidative states, ranging from Cr^2+^ to Cr^6+^ (Rodríguez et al., 2007). Cr (VI) is a hard‐to‐biodegrade metal that can harm the ecosystem and pose a number of health hazards to humans if it is left in soil and water for a protracted period (Sharma et al., 2022). Common applications for the Cr (VI) compounds include the preparation of certain dyes, leather tanning, chrome plating, and wood preservation (Singh et al., 2013). Cr (VI) enters the human body through drinking water and is absorbed in the digestive system. As a result, Cr (VI) accumulates cellularly in a number of important organs, such as the liver, kidneys, brain, and heart, leading to a variety of health disorders (Islam et al., 2022). Moreover, Cr (VI) is the diffusible form of chromium that builds up in the tissues of exposed animals. It causes disruptions of normal cellular integrity and severe disruptions of enzymatic and biochemical processes linked to the metabolic process (Yao et al., 2008). The primary mechanism underlying Cr (VI) toxicity is believed to involve the interaction between elevated levels of Cr (III) and DNA, leading to damage. Within cellular environments, Cr (VI) can undergo reduction to Cr (III), generating reactive intermediate chromium species along with reactive oxygen species (ROS). These ROS, generated through this process, can adversely affect macromolecules, causing lipid peroxidation, DNA damage, and cellular injury, ultimately culminating in necrosis and cell death (Mattia et al., 2004). Extended chromium exposure may lead to nephrotoxicity, potentially becoming the primary cause of death. The kidney, being one of the main organs for chromate accumulation, is particularly susceptible. The buildup of chromate in the vacuoles of proximal tubular cells leads to tubular injury and nephrotoxic effects, ultimately reducing excretion rates and prolonging chromium retention in the kidneys (Hegazy et al., 2016). As heavy metals are typically eliminated via the kidneys, excess Cr (VI) accumulates there, causing nephrotoxicity (Goodarzi et al., 2017). Chromium can affect cellular activity by inhibiting antioxidant enzymes and interacting with antioxidant substances like glutathione, Chromium can have an impact on cellular activity. The production of oxidative stress may provide an explanation for the cytotoxicity induced by heavy metals (Valko et al., 2006). Therefore, nephrotoxicity is mostly related to oxidative stress caused by Cr toxicity, which causes kidney problems and tends to primarily target apoptosis and necrosis as well as mitochondrial damage in the proximal tubular cells (Sun et al., 2015). Because a large number of mitochondria in renal tubule cells produce a lot of energy for solute reabsorption, kidney cells are particularly susceptible to cellular oxidative stress, causing damage (Eirin et al., 2017). Furthermore, high levels of chromium in kidney tissues cause increased ROS generation, which in turn encourages nitrosative and oxidative stress, genomic and cellular damage, and finally leads to necrotic and apoptotic renal cell death (Sahu et al., 2014).

The natural antioxidants found in some fruits, seeds, and natural products have been found to have varying health‐promoting benefits (Rahaman et al., 2023). Many phenolic compounds found in vegetables and fruits are essential dietary components, obligatory for health (Martínez et al., 2012). Medicinal plants include phenolic chemicals, primarily in the form of flavonoids, tannins, and phenolic acids. These substances have numerous antioxidant properties (Martins et al., 2011). *Persea americana*, called avocado, is a palatable fruit from tropical regions and Central America (Leite et al., 2009). Moreover, the avocado seed contains phenolic chemicals such as abscisic acid, furanoic acids, triterpenes, proanthocyanidins, polyphenols, and phytosterols (Ogunka‐Nnoka et al., 2019). Interestingly, ASE had the highest level of activity among the 15 fruit waste extracts tested for antioxidant characteristics (Dao et al., 2016). Avocado seeds are rich in polyphenols, known for their antioxidant and antibacterial properties. Compared to leaves or pulp, avocado seeds contain a significantly higher concentration of phenolic compounds, with a content of 44.89 mg/kg (Antasionasti et al., 2017). In vitro studies have demonstrated the antioxidant effects of avocado seeds, including the inhibition of thiobarbituric acid reactive compounds formation, stabilization of peroxyl radicals and superoxide anions, reduction of Fe^3+^ ions, and inhibition of β‐carotene blanching (Tremocoldi et al., 2018). Additionally, avocado seed extracts exhibit remarkable hydroxyl radical scavenging activity, particularly in the Reed variety, with a value of 13.25 mg AAE/g (Lyu et al., 2023). Furthermore, aqueous extracts of avocado seeds demonstrate antioxidant potential, effectively preventing radical‐induced oxidative damage caused by Fe^2+^ and sodium nitroprusside solutions in rat brains (Oboh et al., 2016). These antioxidant properties may be attributed to the synergistic effects of phenolic components and saponins present in avocado seeds, including phenolic components and procyanidins such as catechin and epicatechin, which contribute significantly to the antioxidant activity of the whole avocado fruit (Wang et al., 2010). The bioactive compounds give the ASE a wide range of therapeutic qualities, such as anti‐inflammatory, antioxidant, antidiabetic, and hypocholesterolemic properties (Lara‐Márquez et al., 2020) and nephroprotective effects (Saad, 2020).

Previous evidence has demonstrated that oxidative stress is often linked to chromium‐induced nephrotoxicity, where oxidative stress primarily targets necrosis and apoptosis to create kidney issues. The current work sets out to assess ASE's antioxidant capacity to shield rabbit kidneys from Cr (VI)'s harmful effects.

## 2 MATERIALS AND METHODS

### 2.1 Avocado seed extract preparation

Samples of ripe Avocado pears (*Persea americana*) were purchased from Cairo, Egypt. Seeds were obtained then minced utilizing a greater, dried, ground to powder, and stored in a glass container. The dried Avocado seeds (100 g) were added to 1 L of hot water in a flask. The avocado seed mixture was filtered after 12 h, and the filtrates were then evaporated in an oven at 40°C to create dried residues (active principles). The aqueous extract was prepared and adjusted to a concentration of 0.4 g/L (Alhassan et al., 2012).

### 2.2 Fourier transforms infrared (FTIR) spectroscopy study

FT‐IR spectroscopy was used to analyze the functional groups of the active components that are present in ASE based on the peak values in the region of IR radiation. Samples were investigated in the range of transmittance (%) scanning between 4000 to 400 cm^−1^ (mid‐IR region) according to the method of Pakkirisamy et al. (2017).

### 2.3 Experimental protocol

Fifteen male rabbits (New Zealand white rabbits) between 700 and 1000 g were obtained from the animal breeding unit of the College of Veterinary Medicine, University of Sohag, Egypt. The rabbits were acclimatized to normal environmental conditions with controlled temperature and humidity, besides they were provided with food and water ad libitum. The study protocol was adjusted in accordance with the guidelines provided by the internal research regulatory and the animal ethics committee for laboratory animal use at Sohag University, Egypt (Faculty of Science: Department of Zoology). Approval for the experimental procedures was granted under the approval number CSRE‐11‐23, adhering to ethical principles.

Chromium toxicity in rabbits was accomplished with 5 mg/kg b.w of K_2_Cr_2_O_7_ (Sigma Aldrich, St. Louis, MO, USA) according to El‐Demerdash et al. (2006). Also, previous studies (Alhassan et al., 2012; Egbuonu et al., 2018) were used to determine the aqueous avocado seed extract dose (400 mg/kg) and treatment time. The rabbits were randomly divided into three equal groups of five rabbits each. The first group made up the control group. The second and third groups received a daily dose of K_2_Cr_2_O_7_ (5 mg/kg) intraperitoneal for 2 weeks. The third group was given a daily oral dose of ASE (400 mg/kg) for 2 weeks.

At the end of the experiment, the rabbits were killed, and blood samples were collected in anticoagulant‐free tubes. The samples were then allowed to stand at ambient temperature for 30 min before being centrifuged at 3500 rpm to separate the serum. Following animal dissection, the entire kidney of the rabbits was promptly removed and cleaned. Half of each kidney was used for histological examination. The other kidney half was weighed and homogenized in the ice‐cold medium. The homogenate was centrifuged at 3000 rpm for 10 min at 4°C. The supernatant was utilized to determine various biochemical parameters.

### 2.4 Oxidative stress markers

Malondialdehyde (MDA) measurement was used to test the oxidative stress in kidney homogenates using a method previously reported by Ramos‐Vara et al. (2008). In brief, MDA and thiobarbituric acid react in an acidic milieu for a duration of 30 min at 95°C to create thiobarbituric acid reactive substances. The wavelength of absorption of the pink product that results can be evaluated at 534 nm.

### 2.5 Antioxidant status

Following the manufacturer's instructions and commercial kit protocols (Diagnostic, Cairo, Egypt), all assessment assays and kits were carried out. Glutathione (GSH) levels as well as SOD, CAT, and GPx activity were measured in kidney homogenates. GSH was measured according to the method described by Beutler et al. (1963). This method was based on the reduction of 2‐nitrobenzoic acid by glutathione, producing a yellow reduced chromogen, which is correlated with the GSH content at 405 nm. Superoxide dismutase (SOD) was determined by using a procedure earlier outlined by Nishikimi et al. (1972) in which the ability of the enzyme to prevent nitroblue tetrazolium dye from being reduced, which is caused by phenazine methosulphate. Measurement of catalase (CAT) was carried out according to the protocol described by Aebi (1984) by using a catalase inhibitor; the catalase reaction with H_2_O_2_ was stopped. The chromophore is produced by the reaction of the residual H_2_O_2_ with 4‐aminophenazone, 3,5‐dichloro‐2‐hydroxybenzene sulfonic acid, and peroxidase in the presence of enzymes. The amount of catalase present in the initial sample is negatively correlated with the intensity of color. The procedure of Paglia and Valentine (1967) was used for quantification of Glutathione peroxidase activity (GPx). This involves the reduced state of oxidized glutathione (GSSG) which is primarily created by the glutathione reductase enzyme reducing organic peroxide (GPx). On the other hand, the presence of hydrogen peroxide starts the oxidation of NADPH to NADP^+^, which is measured by a decrease in absorbance at 340 nm.

### 2.6 Antioxidant enzyme expression

SOD, CAT, and GPx were among the endogenous antioxidant enzymes that underwent quantitative study. The PureLink FFPE RNA isolation kit (Thermo Fisher Scientific, USA) was used to isolate the RNA for this experiment. Four pieces of paraffin‐embedded, formalin‐fixed, 10 m slices of kidney tissue were put into a sterile microcentrifuge tube for RNA extraction, and samples were processed following the manufacturer's recommendations. In short, the tissue was centrifuged free of the melted paraffin and then digested with Proteinase K. The samples were then processed further using the kit's Spin Cartridge, which has a membrane made of silica and is capable of selectively binding RNA. Systematic rinsing with wash buffer removed impurities. In RNase‐free water, the entire RNA was eluted. Using a NanoDrop ND‐1000 spectrophotometer from Nanodrop Technologies (USA), RNA quantity and purity were assessed. For further examination, RNA samples with an OD 260/280 absorbance ratio greater than 2.0 were employed.

Agar gel electrophoresis was used to determine the purity of the RNA, and it was then frozen at −20°C for later use. The primers were designed by utilizing the nucleotide sequence alignment NCBI BLAST program, Blastn (http://blast.ncbi.nlm.nih.gov/Blast.cgi). The primers were used in the following order: CATF 5′‐GGCAAGGTACTTATCGAG; CATR 5′‐TCCACCACCCTTAGGGCTGA; SODF 5′‐AGCTGCACCACAGCAAGCAC; SODR 5′‐TCCACCACCACCCTTAGGGCTGA; GPxF 5′‐GGATTTGGTCGTATTGGG; and GAP DHAR 5′CGACATACTCAGCACCGG. The GAPDH gene served as a housekeeping control for the expression of the gene, with F standing for forward primer and R for reverse primer of the gene. The primers' certificates of analysis were acquired from Macrogen Korea. The reverse primer for each gene was used to create the cDNA. A volume of 20 μL of reverse transcription reaction mix was quickly prepared by utilizing Thermo Fisher Scientific's M‐MLV reverse transcriptase (200 U/L). The reaction mixture was incubated at 42°C for an hour to produce cDNA. The cDNA was kept in reserve for future use at −20°C. Quantitative real‐time PCRs were performed using an Applied Biosystems 7500. The 20 μL total reaction mixture for each sample was made up of 1 μL diluted cDNA, 5 pmol of forward and reverse primers, and 10 μL of 2 SYBR Premix Ex Taq II (Takara Bio Inc., Japan), following Livak and Schmittgen's (2001) instructions for amplification and data processing. In triplicate, each sample was processed. The 2^−ΔΔCT^ method was used to calculate the numerical fold changes in mRNA levels compared to GAPDH mRNA levels.

### 2.7 Histological examinations

Kidney tissues were processed for the typical processes of histological sectioning after being fixed for 48 h. The specimens were dehydrated in ascending series of alcohol, 1 h each; cleared and embedded wax, 1 h each. Sections, 5 μm thick, were cut and mounted on clean glass slides. Harris's hematoxylin and eosin counterstaining were used to stain kidney tissue slides following the previously reported method (Bancroft & Gamble, 2008). A semi‐quantitative score was used to measure kidney injury, which is based on a percentage of glomeruli shrinkage and atrophy, tubulointerstitial damage, loss of tubular brush border, necrosis, and vacuolar degeneration in renal tubules as well as hemorrhage, congested blood vessels, and cellular infiltration [score 0 = no damage; 1 = ≤10%, 2 ≤ 11%–25%, 3 ≤ 50%, 4 ≤ 75%, and 5 > 76% damage] as described before (Kaur et al., 2021).

### 2.8 Histochemical examinations

Other slides were stained for collagen fiber distinction with Masson's trichrome dye, as was reported earlier (Avwioro, 2011). The Masson trichrome stained area was delineated and quantified using ImageJ software, as reported by Hernández‐Morera et al. (2016). For carbohydrate detection, the periodic acid Schiff's (PAS) was used as was reported earlier (Meyerholz et al., 2018). Then sections were dehydrated in ascending grades of ethanol, cleared in xylene, and mounted with DPX.

### 2.9 Immunohistochemistry examination

Cyclooxygenase‐2 (COX‐2) was detected in 4 m thick deparaffinized kidney tissue slices. Briefly, anti‐COX‐2 antibodies diluted 1:100 were treated overnight with deparaffinized slices. Endogenous peroxidase activity was then suppressed by incubating the slices with 0.075% hydrogen peroxide in PBS. A peroxidase/DAB kit was used for the DAKO EnVision+ System to identify antibodies. The sections were then mounted, dehydrated, and counterstained with hematoxylin. By using light microscopy, the intensity of the COX‐2 immunostaining and its cellular location were examined.

The mean area percent of collagen fiber, PAS‐positive reactions, and COX‐2 positive reactions were measured in 10 non‐overlapping high‐power fields (40) of paraffin sections of kidneys in each group using image analysis software (ImageJ version 1.46, NIH, USA) and statistically analyzed.

### 2.10 Statistical analysis

The organization and analysis of the data was done using GraphPad Prism® version 5.01 (GraphPad Software Inc., San Diego, CA, USA). The means ± SE are used to express the results. A one‐way analysis of variance (ANOVA) was used to statistically analyze the results followed by Bonferroni's multiple comparison test. Significant (*p* values) were defined as those ≤.05.

## 3 RESULTS

### 3.1 FT‐IR spectra

**FIGURE 1:**
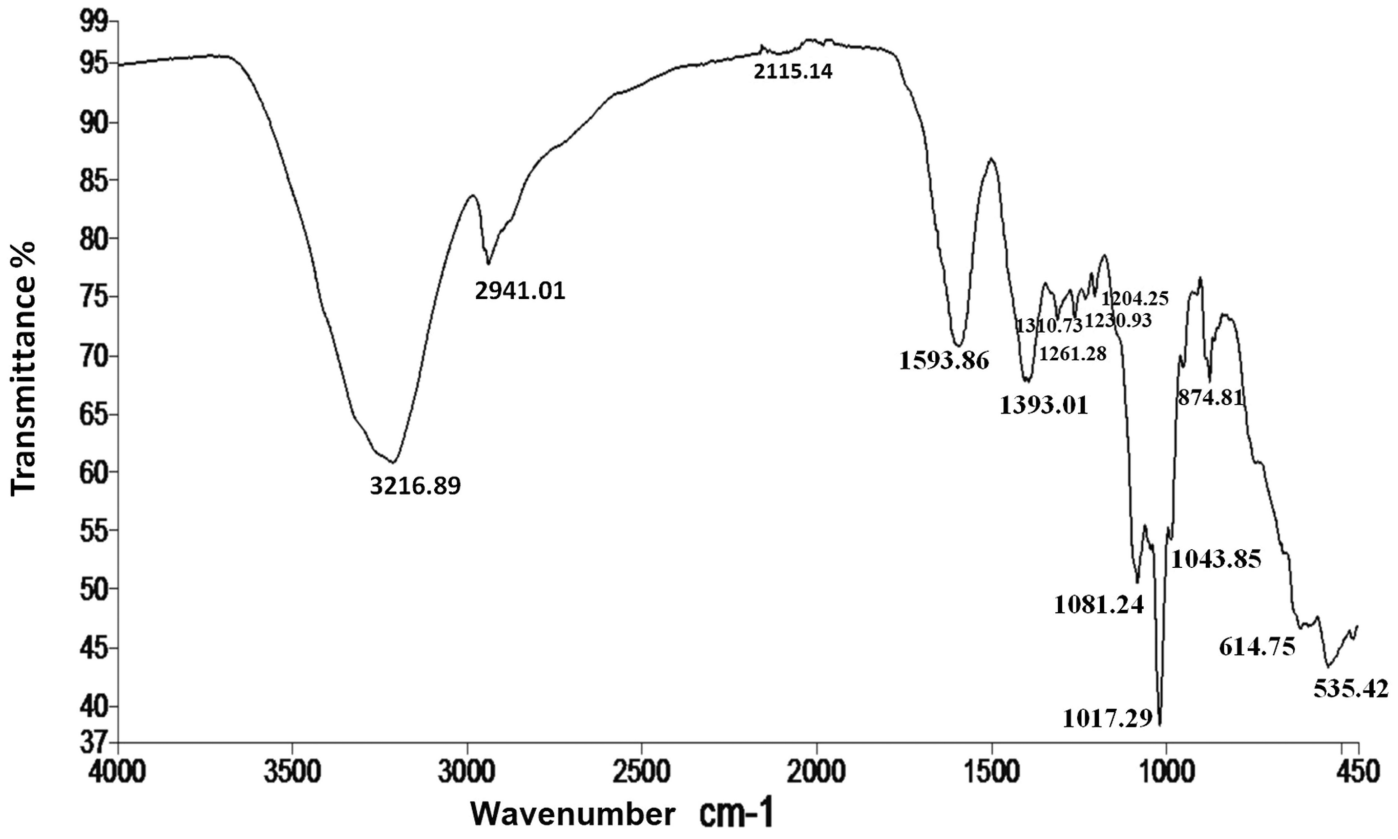
Infrared spectroscopy of aqueous avocado seed extract (ASE).

Figure 1 and Table 1 demonstrate the IR spectrum analysis of avocado seed extract. However, there are just a few papers on infrared analysis of avocado seed extract. The majority of the frequencies are group frequencies, which indicate whether or not a sample contains particular functional groups. The aqueous seed extract of Avocado is characterized by the presence of OH, CH, C‐N, C‐F, C C‐Br, ‐Cl, C‐O, CO‐O‐CO, C=O, C=C, S=O groups. As a result, it may be deduced that amine, aromatic or aliphatic alcohols, halo‐compound and fluoro‐compound, and Sulfonyl chloride compounds are among the components of the extract. The FTIR spectra of ASE show a wide absorption peak at 3216.89 cm^−1^, indicating the vibration of −OH and −NH stretching bonds associated with the presence of alcohols, phenols, carboxylic acids, and primary/secondary amines. The weak peak observed at 2115.14 cm^−1^ is linked to the existing C≡C stretching and is ascribed to the existence of alkynes. The primary and aromatic amines are represented by the wavelength of absorption at 1539.88 cm^−1^, which are attributed to the −NH bending and C−N stretching bonds (Ngungeni et al., 2023). The wavelength of absorption peaks at 1393.01 and 1310.73 cm^−1^ indicating the vibration of S=O stretching bonds which is associated with the presence of sulfonyl chloride compounds. The absorption peaks at 1261.28, 1230.93, and 1204.25 cm^−1^ indicating the vibration of C‐O stretching bonds associated with the presence of Vinyl and Alkyl aryl ethers compounds. The bands at 1081.24.60, 1043.85, and 874.81 cm^−1^ are corresponding to C‐O, CO‐O‐CO C‐H stretching vibration of the aromatic ring. ASE exhibited an absorption peak at 1017.29, 614.75, and 535.42 cm^−1^ attributed to a C‐F stretching, C−Br, and C‐Cl stretching, respectively, associated with the presence of fluoro compound and halo compound. Previous study has shown maxima at 3330, 3000–2840, 2920, 1730, 1580, and 1150–1000 cm^−1^ in raw avocado seeds (Fregue et al., 2019).

**TABLE 1:** IR spectrum of avocado extract by frequency range.

Absorption (cm^−1^)	Appearance	Transmittance (%)	Group	Compound class
3216.89	Strong, broad	13.49	O‐H stretching	Alcohol
2941.01	Medium	12.33	C‐H stretching	Alkane
2115.14	Weak	8.87	C≡C stretching	Alkyne monosubstituted
1593.86	Medium	6.68	N‐H bending	Amine
1393.01	Strong	5.84	S=O stretching	Sulfonyl chloride
1310.73	Strong	5.49	S=O stretching	Sulfone
1261.28	Strong	5.29	C‐O stretching	Vinyl ether
1230.93	Strong	5.16	C‐O stretching	Alkyl aryl ether
1204.25	Strong	5.05	C‐O stretching	Vinyl ether
1081.24	Strong	4.53	C‐O stretching	Primary alcohol
1043.85	Strong, broad	4.37	CO‐O‐CO stretching	Anhydride
1017.29	Strong	4.26	C‐F stretching	Fluoro compound
874.81	Strong	3.67	C‐H bending	1,2,4‐trisubstituted
614.75	Strong	2.57	C‐Br stretching	Halo compound
535.42	Strong	2.24	C‐Cl stretching	Halo compound

### 3.2 Oxidative stress markers

The MDA levels in the kidney homogenates of rabbits in all experimental groups are presented in (Figure 1a). Cr (VI) administration caused a significant elevation (*p* < .001) in renal MDA level (69.3 ± 4.1 nmol/g) compared to the untreated rabbits group (6.8 ± 1.3 nmol/g). However, the administration of ASE with Cr (VI) attenuated the MDA level (24.5 ± 2.7 nmol/g) significantly (*p* < .001) compared to the rabbits receiving Cr (VI).

### 3.3 Antioxidant status

**FIGURE 2:**
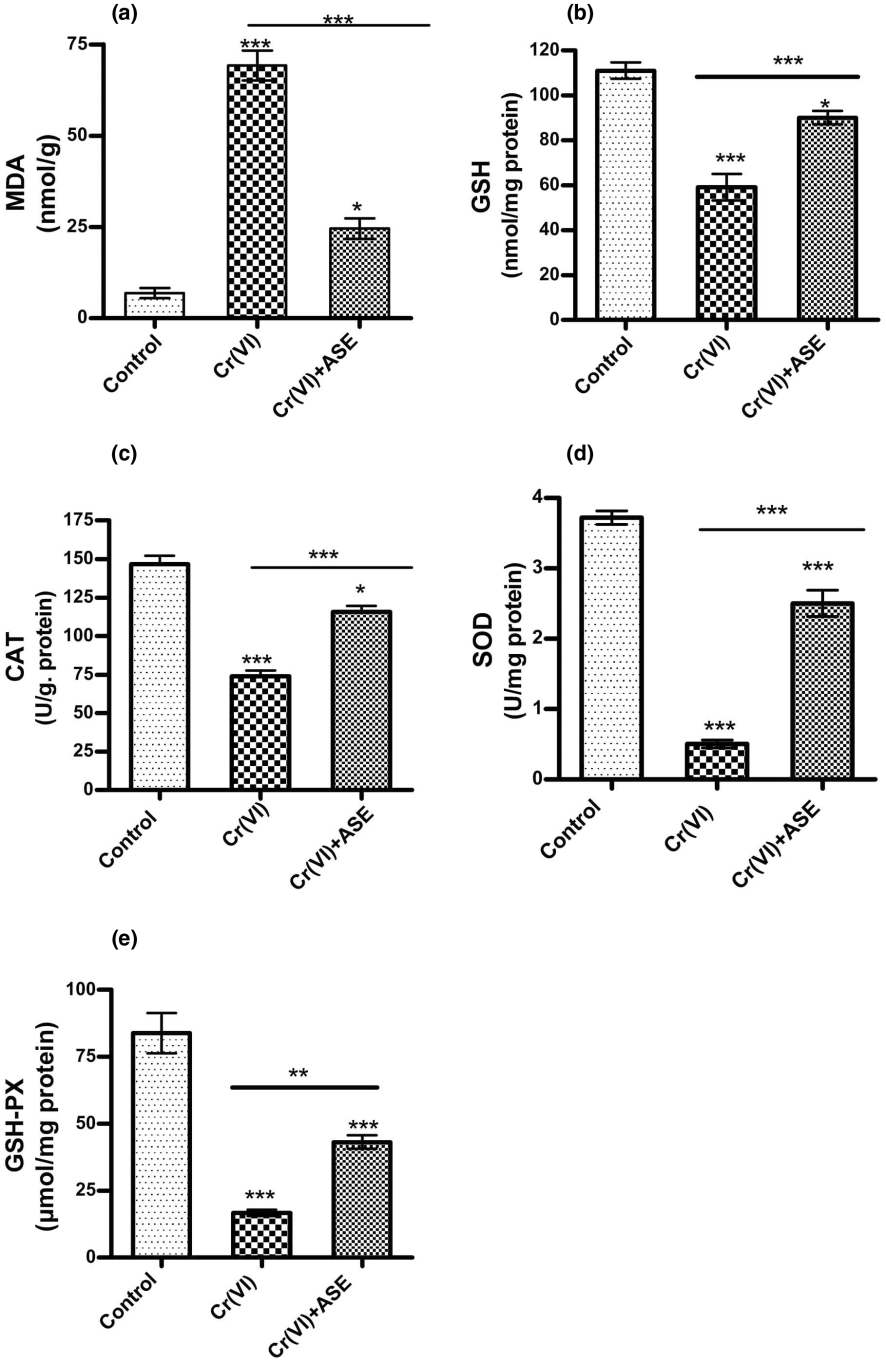
Effect of avocado seed extract (ASE) on MDA (a) and GSH (b) levels and the antioxidant enzyme activities; SOD (c), CAT (d), and GPx (e) in kidney tissue of Cr (VI)‐treated rabbits. The number of rabbits was 5. Significance: *p* < .05 (*), *p* < .01 (**), *p* < .001 (***).

The Cr (VI)‐driven oxidative stress in renal tissue was manifested by a highly significant reduction (*p* < .001) GSH content (59.16 ± 5.89 nmol/mg) in the kidney for Cr (VI)‐treated rabbits as compared to that of the control group (111.1 ± 3.63 nmol/mg). This decline in the GSH level was offset by the supplementation of the Cr (VI)‐treated rabbits with ASE (90.16 ± 2.98 nmol/mg) (Figure 2b). Likewise, administration of Cr (VI) led to a highly significant reduction (*p* < .001) in the activities of SOD (0.5 ± 0.0505 U/mg protein), and GPx (16.76 ± 1.11 μmol/mg protein) and a substantial reduction (*p* < .05) in the activity of CAT (73.84 ± 3.91 U/g protein) compared to the levels in the control group which recorded 3.72 ± 0.09 U/mg protein, 83.88 ± 7.5 μmol/mg protein, and 146.7 ± 5.33 U/g protein respectively (Figure 2c–e). The co‐ administration of ASE with Cr (VI)‐treated rabbits dramatically recovered the SOD, CAT, and GPx activities which recorded 2.5 ± 0.18 U/mg protein, 115.7 ± 4 U/g protein, and 43.22 ± 2.51 μmol/mg protein respectively in kidney tissue, compared to the Cr (VI) group (Figure 2c–e).

### 3.4 Antioxidant gene expression

**FIGURE 3:**
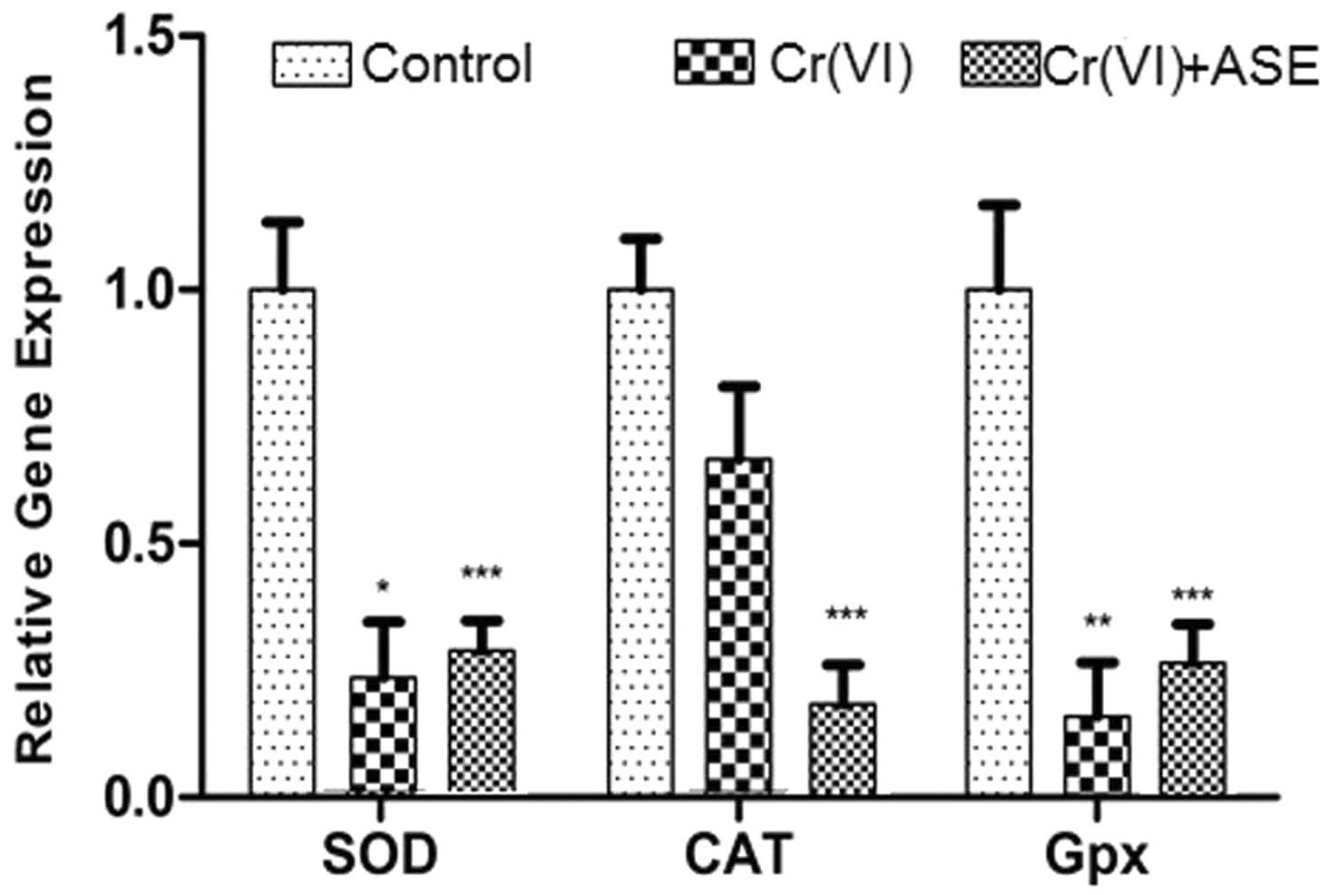
The effects of aqueous avocado seed extract on gene expressions of antioxidant enzymes SOD, CAT, and GPx in kidney tissue of rabbits and Cr (VI) + ASE group represents data for rabbits treated with Cr (VI). Significance: *p* < .05 (*), *p* < .01 (**), *p* < .001 (***).

The expression of the SOD, CAT, and GPx genes was significantly (*p* < .001) lower in the Cr (VI)‐treated rabbits than those in the untreated group (Figure 3). On the other hand, group 3's ASE supplementation demonstrated a substantial upregulation of the SOD and GPx expression (*p* < .05) in comparison to the Cr (VI)‐treated rabbits. On the contrary CAT expression was still reduced when Cr (VI) was co‐administered with ASE compared to rabbits treated with Cr (VI).

### 3.5 Histological investigations

**FIGURE 4:**
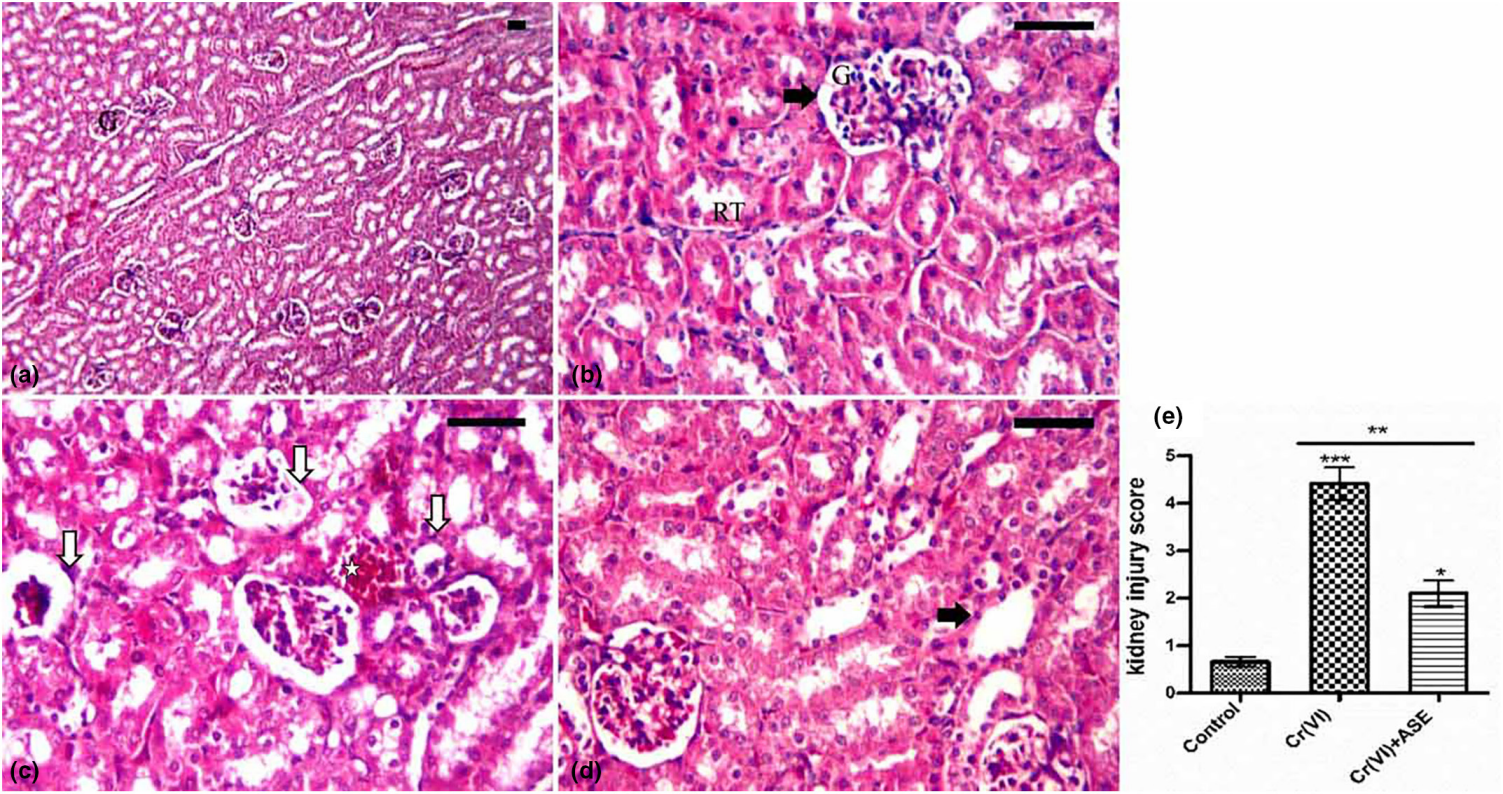
Photomicrographs from kidney tissues collected from rabbits of control, Cr (VI), and ASE + Cr (VI) groups stained with hematoxylin–eosin. (a, b) Kidney tissue of the control group showed normal histological structure of the kidney cortex showing the glomerulus (G), Bowman's space (arrow), and Normal renal tubules (RT). (c) Kidney tissue of the Cr (VI)‐treated group showed shrinkage and atrophy (white arrow) of most of the Glomeruli, an increase in the Bowman's space, extensive vacuolar degeneration in renal tubules, and swollen epithelial lining cells in some tubules. There was hemorrhage, congested blood vessels, and cellular infiltration in some areas (*). (d) Renal tissue of ASE + Cr (VI)‐treated group resulted in some glomeruli (G) and some of the renal tubules swelled (arrow). Scale bar: 50 μm Analysis of live injury score (e). Significance: *p* < .05 (*), *p* < .01 (**), *p* < .001 (***).

The histological structure of the kidney cortex of rabbits in the control group stained with H&E showed normal renal tubules and renal corpuscles with normal Bowman's space and glomeruli. (Figure 4a,b). The renal architecture in Cr (VI) treatment rabbits showed severe histopathological changes, including extensive vacuolar degeneration in renal tubules and swollen epithelial lining cells in some tubules, shrinkage and atrophy of most of the Glomeruli, and an increase in the Bowman's space. There was hemorrhage, congested blood vessels, and cellular infiltration in some areas (Figure 4c). Treatment of rabbits with ASE plus Cr (VI) somewhat reversed the renal injury induced by Cr (VI) (Figure 4d). The renal tissue remained an intact architecture with less degeneration areas than those observed in Cr (VI) alone.

The scoring of kidney injury in the kidney sections revealed significant damage (*p* < .001) in the rabbits receiving Cr (VI) in the form of Glomeruli shrinkage and atrophy, loss of tubular brush border, tubulointerstitial damage, necrosis, and vacuolar degeneration in renal tubules as well as hemorrhage, congested blood vessels, and cellular infiltration in renal tissues compared to the control group. On the other hand, the treatment of ASE with Cr (VI) prevented renal injury and resulted in a substantial (*p* < .05) reduction in kidney injury score compared to the rabbits treated with Cr (VI) (Figure 4e).

### 3.6 Histochemical examinations

#### 3.6.1 Masson trichrome‐stained tissues

**FIGURE 5:**
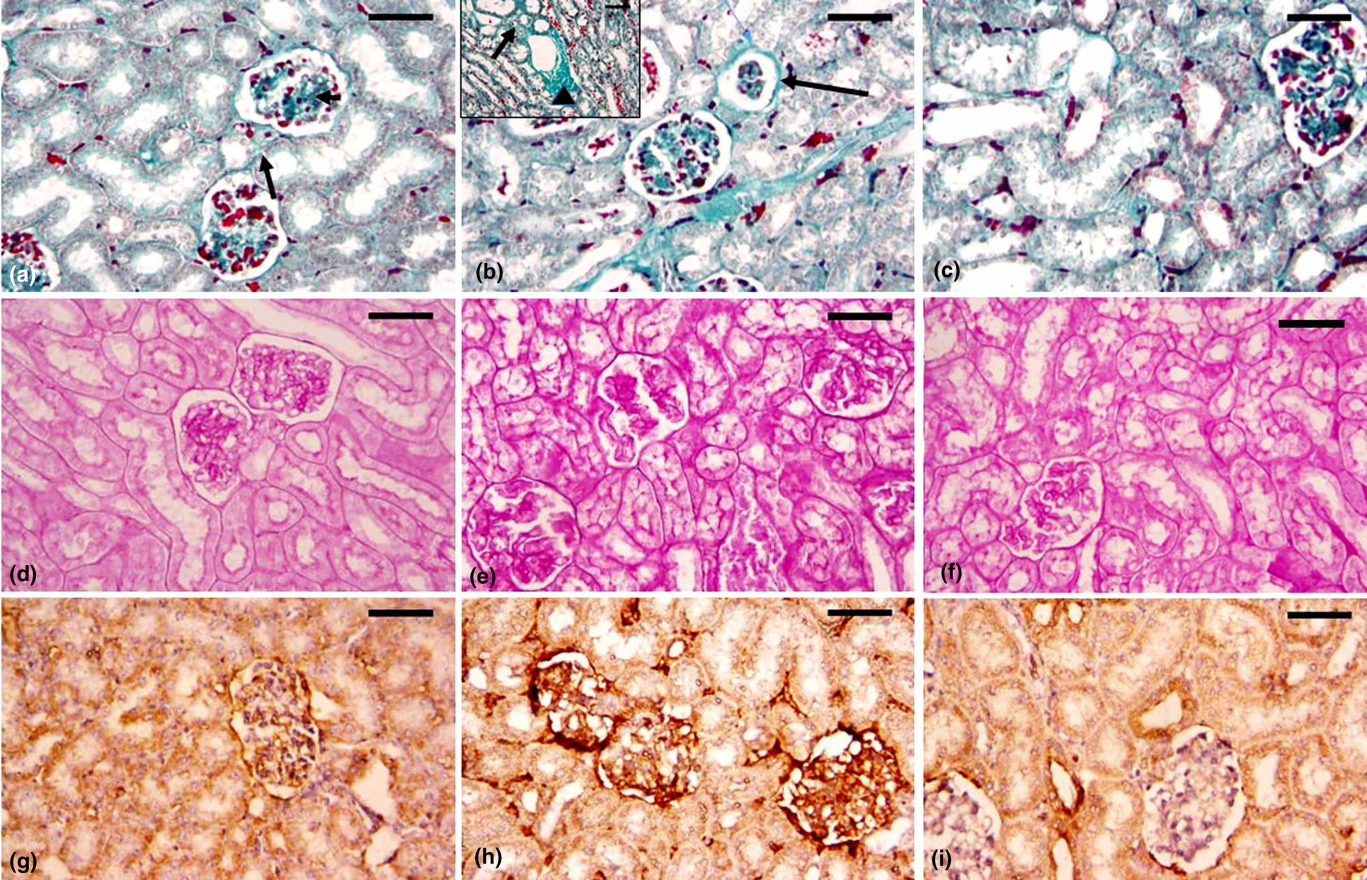
Photomicrographs from kidney tissues collected from rabbits of control, Cr (VI), and ASE + Cr (VI) groups stained with Masson Trichrome stain (a–c). (a) Renal from the control group showing scanty interstitial connective tissue and scanty collagen content surrounding the glomeruli. (b) Cr (VI)‐treated rabbits showed a marked increase in the collagenous content in the interstitial connective tissue (white arrow), in the peri‐glomerular (long arrow), around the blood vessels (arrowhead), and the intertubular spaces (short arrow). (c) The ASE + Cr (VI) group showed a somewhat moderate decrease in the collagen fibers as compared to those observed in the Cr (VI) group. Scale bar 50 μm. Photomicrographs of PAS‐stained kidney sections (d–f). (d) the control group showing the normal PAS positivity in the glomeruli and the brush border of kidney tubules. (e) Cr (VI)‐treated group showing the elevation of carbohydrate contents in the glomeruli and kidney tubules. (f) The Cr (VI)‐treated group with ASE showed improvement in carbohydrate contents as compared to the Cr (VI)‐treated group. Scale bar 50 μm. Photomicrographs (g–i) of kidney tissues stained with COX‐2 immunostaining. (g) Kidney tissue of the control rabbits showed on‐detectable levels of COX‐2 expression. (h) Kidney tissue of the Cr (VI)‐treated group showing intensive and widespread reaction for positive immunoreactivity for anti‐COX‐2 indicated by dense brown staining in the renal tissue. (i) Renal tissue of ASE plus Cr (VI)‐treated rats showing a marked decrease in the COX‐2 amount compared to the Cr (VI) group alone. Scale bar 50 μm.

**FIGURE 6:**
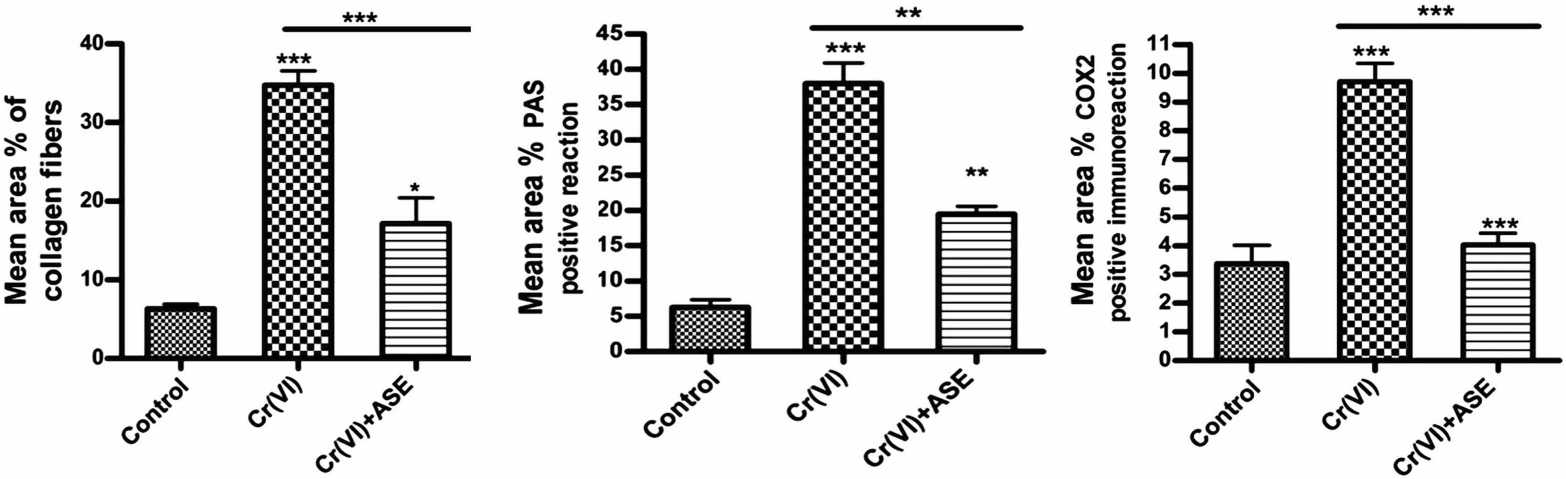
Quantitative analysis of mean area percent of mean area percent of collagen fiber, PAS‐positive reactions, and COX‐2 positive reactions in the kidney tissue of rabbits in experimental groups. Values are mean ± SE. Significance: *p* < .05 (*), *p* < .01 (**), *p* < .001 (***).

Masson trichrome staining of control renal tissues revealed little interstitial connective tissue focused primarily around blood veins. Scanty collagenous content around the glomeruli and peritubular zones was observed (Figure 5a). The renal tissue of rabbits treated with Cr (VI) showed a marked increase in the collagen fiber content around the renal corpuscles and the blood vessels as well as in intra‐glomerular and intertubular spaces (Figure 5b). The renal tissue of ASE plus Cr (VI)‐treated rabbits showed a somewhat moderate decrease in the collagen fibers as compared to those observed in Cr (VI) alone (Figure 5c). The area percent of collagen fibers in the Cr (VI)‐treated animals was substantially higher (*p* < .001) than those in the control group. When compared to the Cr (VI)‐treated group, ASE‐treated rabbits had a significantly lower collagen fiber area percent (*p* < .05) (Figure 6a).

#### 3.6.2 PAS‐stained tissues

Histochemical examination of PAS‐stained kidney tissues of the control group showed normal PAS‐positive reaction in the brush border of renal tubular cells and glomeruli (Figure 5d). Cr (VI)‐administrated group showed an increase in PAS‐positive reaction in the kidney tubular brush border and the glomeruli (Figure 5e). The co‐administration of ASE with Cr (VI) showed improvement in the stainability with PAS that looks similar to the control as compared to Cr (VI)‐treated group (Figure 5f). The area % of PAS‐positive reactions increased significantly (*p* < .001) in the Cr (VI)‐treated group compared to the control group. In the ASE with Cr (VI)‐administrated rabbits, a substantially decreased (*p* < .05) PAS‐positive reactions area percent was seen as compared to the rabbits treated with Cr (VI) (Figure 6b).

#### 3.6.3 Immunohistochemical analysis

The immunohistochemistry analysis in renal tissue of the control group showed undetectable levels of COX‐2 expression (Figure 5g). When compared to the rabbits in the control group, the COX‐2 expression level in the kidney's tubules and glomeruli significantly increased in the Cr (VI) rabbits (Figure 5h). While the treatment of rabbits with ASE and Cr (VI) showed a decrease in COX‐2 expression in the renal tubules and glomeruli of kidney as compared to the rabbits receiving Cr (VI) (Figure 5i). In comparison to the control group, there was a substantial increase (*p* < .001) in the area percent of COX‐2 expression. ASE‐treated rabbits showed a considerably lower COX‐2 expression area percent (*p* < .05) than the Cr (VI)‐treated group (Figure 6c).

## 4 DISCUSSION

The kidneys play a crucial role in regulating numerous substances, highlighting their significance in maintaining public health. Renal pathogenesis poses a significant concern, particularly in cases where there is a deficiency in the endogenous antioxidant defense mechanism. It has been demonstrated that the antioxidant activity of medicinal herbs lowers ROS and increases the antioxidant defense system, therefore reducing oxidative kidney injury.

The findings of our study revealed that Cr (VI) exposure led to a noteworthy increase in MAD levels, as well as a decline in GSH and the activities of antioxidant enzymes (GPx, SOD, and CAT). However, Cr (VI) promotes oxidative stress in the cell membrane, which produces free radicals, which have a lot of harmful effects inside the cells and damage renal tissue (Bagchi et al., 2002). An imbalance between antioxidants and oxidants promotes the generation of ROS, which can lead to lipid peroxidation, gene expression change, DNA modification, and ultimately cell death (Parveen et al., 2009).

The outcomes are in line with previous studies of nephrotoxicity caused by Cr toxicity as a result of an increase in ROS, a disruption in the antioxidant balance, and chromosomal changes in rats (Chakraborty et al., 2022). Also, Khalaf et al. (2020) demonstrated a substantial rise in MDA levels along with a decline in GSH levels as well as the activity of SOD and CAT enzymes in renal tissue homogenates of the Cr‐received rats. Cr (VI) toxicity is directly connected to dosage and duration, where prolonged Cr (VI) exposure increases cellular oxidative stress, causing death in cells (Xu et al., 2018), and also leads to the reduction of tissue nonenzymatic and enzymatic antioxidants (GSH, CAT, and SOD) (Boşgelmez et al., 2008) as an adaptive response to oxidative stress (Soudani et al., 2010). According to Soudani et al. (2012), the decrease in GSH level and enzymatic antioxidants (SOD and CAT) activities in renal tissue may be caused by its use in the scavenging of free radicals during chromate detoxification. Rats exposed to Cr also displayed elevated ROS levels with renal superoxide anions and hydrogen peroxide production (Sahu et al., 2014). Additionally, GSH formation is constrained during Cr (VI) metabolism due to the oxidation of the precursor thiol amino acid, which lowers GSH levels (Goodarzi et al., 2017). The activity of all three enzymes is constrained in renal tissue where the decreased antioxidants in the cell can cause endogenous ROS accumulation that stimulates signaling paths leading to cellular death in renal tubules (Boşgelmez & Güvendik, 2017). Accordingly, the presence of Cr (VI) altered the CAT structure to varying degrees, which may also have an impact on the functions of other enzymes and result in a reduction in CAT activity throughout all organs (Chen et al., 2018). Additionally, exposure to Cr compounds causes oxidative stress and tissue damage by increasing the kidney's ROS levels (Zheng et al., 2020).

The gene expression data for the enzymes SOD, GPx, and CAT decreased significantly in kidney tissue after Cr (VI) injection. According to Lushchak et al. (2008), an elevated formation of ROS may be the primary factor contributing to Cr (VI) toxicity, which can interact with proteins and DNA, causing changes and defects in chromosome structure as well as inhibiting antioxidant enzyme action. On the other hand, ROS could inhibit the transcription of antioxidant enzymes by acting at the genomic level. Thus, by decreasing the activity and transcription of enzymes, our results further demonstrated that the generation of ROS affects both the cellular and genomic levels (Al Mahmud et al., 2017; Feng et al., 2017). Furthermore, the alteration of transcription regulation is the main source of Cr (VI) toxicity, making normal gene expression problematic (Pavesi & Moreira, 2020). Cr (VI) stimulates transcriptional downregulation of genes implicated in the antioxidant pathway while upregulating the apoptosis‐related genes caspase (3 and 9) and p53 (Li et al., 2022). In accordance with a recent study, oxidative‐mediated toxicity caused by the conversion of Cr (VI) to Cr (III) can harm organelles inside cells and cause DNA changes (Singh et al., 2023).

In the present work, the upsurge in oxidative stress markers and the inhibition in the activities of antioxidant enzymes caused kidney histopathological alterations in the Cr (VI)‐treated group as manifested by cytoplasmic vacuolation, shrinkage, and atrophy of most of the Glomeruli, an increase in Bowman's space, congestion, hemorrhage, and inflammatory cell infiltration. Morphometry confirmed this, showing a marked rise in the number of renal corpuscles impacted. The present results agree with those of Khalaf et al. (2020), who attribute the harmful effects of Cr to striking pathological changes in kidney tissue brought on by the production of ROS as a result of oxidative stress. However, the kidney is a significant target organ for various toxic substances, and the proximal tubular epithelium inside the kidney is the most prominent target location for cell destruction induced by toxicants (Bashandy et al., 2016). According to studies, a single 15 mg/kg dose of K_2_Cr_2_O_7_ caused histopathological changes in renal tissues, accompanied by significantly increased oxidative stress, apoptosis, and inflammation (Sahu et al., 2014). Also, exposure to Cr compounds resulted in chromatin migration, nucleus shrinkage, loss of cristae, variable mitochondrial size, and observable alterations in certain renal tubules in kidney tissue (El‐Mahalaway et al., 2015). Additionally, kidney tissue exposed to Cr saw alterations in the outer mitochondrial and nuclear membranes, chromatin structure, endothelial cell structure, and pedicles (Venter et al., 2017). Also, Cr (VI) in a dose‐dependent manner generates kidney damage through the alteration of renal ultrastructure and histology, apoptosis, the stimulation of oxidative stress, and disruption of mitochondrial function (Zheng et al., 2020). Similarly, as a result of Cr exposure, the mitochondria became fractured and unevenly shaped, with reduced kidney tissue in size (Avila‐Rojas et al., 2020). After injectable treatment, chromium compounds are specifically deposited in the proximal renal tubules, causing severe tubular necrosis in high doses (Hassan et al., 2021). According to a recent study, the administration of chromium caused alterations in renal tissues such as necrotic renal cells and renal tubular dysfunction in male Wistar rats (Ghosh et al., 2023).

Our study showed the co‐administration of Cr (VI) and ASE effectively mitigated oxidative stress and halted the reduction in antioxidant enzyme expression and activity, which was mirrored in an improvement in kidney tissues' histological structure. In addition, CAT expression was still reduced. Other findings suggest that the ASE can protect the kidneys by preventing oxidative stress in the kidneys (Abdel‐Moneim et al., 2017; US et al., 2014). Moreover, avocado flavonoids may inhibit the development of renal calculi by limiting the generation of ROS, thereby preventing oxidative stress damage to renal cells (Anshar et al., 2018). Also, the nephroprotective efficacy of the ethyl acetate extract of ASE against nephrotoxicity caused by acetaminophen was assessed using a dose‐dependent approach (Ogunka‐Nnoka et al., 2019). According to research by (Elmoslemany et al., 2021), co‐administration with ASE powder reduces oxidative stress damage to the kidney and improves kidney function, structure, and antioxidant status. The results suggest that consuming ASE may have some therapeutic effects on rats' renal impairment caused by cisplatin. The antioxidant capabilities of ASE may be attributed to its phytochemical components as well as its phenolic constituents (Padilla‐Camberos et al., 2013). It is well known that phenolic compounds—polyphenols, flavonoids, fatty acids, tannins, carotenoids, tocopherols, and tocotrienols—belong to the group of lipophilic antioxidants (Sun et al., 2020). In addition, many antioxidants, including endogenous, exogenous, polyphenolic, and enzymatic antioxidants, are part of the cellular defense responses to Cr (VI)‐induced ROS (Asatiani et al., 2010). Medicinal plant‐derived antioxidants enhance the body's own antioxidant defenses, thus mitigating kidney injury by decreasing lipid peroxidation (Rafieian‐Kopaei, 2013). These antioxidants play a crucial role in significantly reducing the generation of reactive oxygen species/reactive nitrogen species (ROS/RNS), which are associated with oxidative stress‐induced kidney damage (Rafieian‐Kopaei, 2013). Recent research suggests that elevated dietary total antioxidant capacity scores yield beneficial effects on renal function (Moludi et al., 2022).

Our current data indicate that co‐administration of ASE and Cr (VI) results in decreased CAT protein expression and increased CAT activity. This could be due to the modulation of catalase activity by post‐translational modifications, such as phosphorylation or acetylation. Accordingly, the antioxidants may affect these modifications, leading to increased catalase activity without a corresponding increase in protein expression. The modifications in the cellular redox potential lead to substantial regulation of the existence or activity of antioxidant enzymes in cells through transcriptional, translational, and post‐translational pathways (Evans et al., 2002). An additional explanation for increased CAT activity in conjunction with decreased CAT protein expression could be a compensatory mechanism aimed at mitigating the adverse consequences of chromium‐induced poisoning. These strategies can include catalase activity upregulation by methods other than enhanced gene expression (Sadi et al., 2014).

Regarding kidney fibrosis, stimulated fibroblasts represent the primary producers of both type I and type II collagen in the perivascular region and the microvasculature after interacting with invasive immune cells and endothelial cells (Jin et al., 2022). In this study, Masson's trichrome stain sections revealed noticeably deposited collagen fibers both within and between the renal tubules in the kidneys of the Cr (VI)‐treated rabbits. This was confirmed by a morphometric study, which revealed that the mean area percent of collagen fibers was much higher than the control. Similarly, it has been shown that Cr (VI)‐induced renal damage in rats led to severe renal fibrosis (Baiomy et al., 2023; Ghosh et al., 2023). Conversely, administering ASE to the Cr (VI)‐treated rabbits led to a substantial reduction in collagen fibers. Furthermore, the polyunsaturated fatty acids and antioxidants in avocado fruit can reduce renal fibrosis and tubular injury through anti‐fibrotic and anti‐inflammatory mechanisms (Shalaby et al., 2020). It has been reported that avocado seed has a number of active ingredients, including saponin and flavonoids, both of which have anti‐inflammatory properties. Saponins inhibit the work of the cyclooxygenase enzyme by catalyzing the reaction of arachidonic acid to endoperoxidase. The cyclooxygenase and lipoxygenase enzyme inhibitions decrease the inflammatory response and accelerate the transforming growth factor‐β (TGF‐β) (Tugiyanti et al., 2019). TGF‐β is a protein with three isoforms (TGF‐β1, TGF‐β2, and TGF‐β3) that plays an important function in renal fibrosis through induction of epithelial‐to‐mesenchymal transition (EMT) (Lee et al., 2015). As a result, TGF‐β1 activation promotes profibrotic genes and causes renal fibrosis, whereas TGF‐β1 inhibition may reduce kidney damage and fibrosis. A recent study indicates that flavonoids could potentially compete with TGF‐β1's ligand‐binding site, indicating that these chemicals should be further investigated for the creation of possible treatments for kidney fibrosis (Rahman et al., 2022).

Cr (VI) is a potential free radical producer as well as a powerful disruptor of metabolic processes in many exposed tissues of organisms (Sridevi et al., 1998). Metabolic dysfunction is one of the most common manifestations of multi‐organ chromium (VI) toxicity. Metabolic dysfunction is one of the most common manifestations of multi‐organ chromium (VI) toxicity. However, Cr (VI) exposure produces cellular infiltration in hepatic and renal tissue due to abnormalities in glucose and protein metabolism. The present histochemical observations of PAS‐stained kidney slides of the Cr (VI)‐treated group showed an increase in PAS‐reaction in the glomeruli and the brush border of kidney tubular cells. A prior study found considerable accumulation of Cr (VI) in renal tissue after short‐term Cr (VI)‐induced toxicity, which was linked to renal metabolic and physiological disorders (Shil & Pal, 2018). Cr (VI) is extremely nephrotoxic and can lead to severe kidney destruction, cellular dysfunction, inadequate renal reabsorption, glycosuria, and proteinuria (Li et al., 2016). In line with our results, dichromate injection caused glucosuria, which results in renal tubule cell destruction (Khan et al., 2010). This can lead to acute kidney disease, which shows visible nephron cell death and renal physiological abnormalities (De Geus et al., 2012). Our study found that combining Cr (VI) with ASE greatly lowers PAS‐stained kidney tissues. It has been reported that the ASE significantly reduced blood sugar levels and restored the histopathological damage to various rat tissues, including the liver, kidneys, and pancreas, in alloxan‐induced diabetic rats (Ezejiofor et al., 2013).

Avocado seeds include secondary metabolites such as quercetin, tannin, rutin, kaempferol, triterpenoids, alkaloids, and saponin, which reduce hyperglycemic conditions through several mechanisms, such as maintaining glucose homeostasis and preventing glucose absorption in the digestive tract. Blood glucose levels can be reduced by inhibiting carbohydrate‐hydrolyzing enzymes, such as α‐amylase and α‐glucosidase. α‐amylase catalyzes the hydrolysis of starch into glucose polymers such as α‐(1,4)‐D‐glycosidic linkages, while α‐glucosidase catalyzes the last stage of the corresponding mono‐ and disaccharide digesting process (Sales et al., 2012). However, the inhibitions of α‐amylase by flavonoids vary with the number, nature, and position of the substituents in the derivative flavonoid structure (Proença et al., 2019). Dihydroxy flavonols are a type of flavonoid that can inhibit α‐amylase enzymes. Flavonoids inhibit α‐amylase by interacting with its active site through OH groups (AK et al., 2019). The antidiabetic activity of secondary metabolites from avocado seeds can also be explained by the presence of triterpenoids, which block the sodium glucose transporter 1 (SGLT1) (Khathi et al., 2013). SGLT1 is responsible for glucose absorption in the small intestine (Gorboulev et al., 2012), and for reabsorbing nearly 3% of the filtered glucose load in the renal proximal tubule (Rieg et al., 2014). In addition to this, SGLT2, a transporter protein, is responsible for the most (90%) of glucose reabsorption from the glomerular filtrate (Gerich, 2010). Also, tannins as an antioxidant have a protective function to ward off free radicals and activate antioxidant enzymes to support the growth, regeneration, and protection of the pancreas (Indrakusuma et al., 2021).

There is mounting evidence linking oxidative stress to the inflammatory response, whereby any alteration in the structural integrity of tissues causes inflammation, which activates a number of repair processes to help the tissue recover to normal (Goldszmid & Trinchieri, 2012). Cyclooxygenase‐2 (COX‐2) is a pro‐inflammatory cytokine that is created by the kidney's proximal tubular cells. However, COX‐2 is stimulated by inflammatory mediators and is believed to be crucial for pathophysiologic processes such as inflammation, tumorigenesis, and angiogenesis (Smith & Langenbach, 2001). So, the immunohistochemical detection of COX2 is another piece of evidence for the chromium toxicity of the kidney tissue. This study showed that Cr (VI)‐impaired kidney injury caused a dramatic rise in COX‐2 expression in the kidney tissue. Previous investigations demonstrated inflammatory alterations due to Cr (VI)‐induced COX‐2 increases in brain and lung tissues (Salama et al., 2016) and spleen tissue (Venter et al., 2020), as well as in a chicken hepatocellular carcinoma cell line (Liu et al., 2020). Conversely, the etiology of nephrotoxicity is significantly influenced by COX‐2 along with other mediators of inflammation. Therefore, reducing COX‐2 expression significantly in the kidneys under oxidative stress may be one of the most important treatment strategies for treating nephrotoxicity (Morsy et al., 2013). Our results exhibited that co‐administration of ASE with Cr (VI) significantly reduces COX‐2 expression levels in kidney tissues. Our outcomes support the idea that exogenous antioxidants that are not enzymes can pass through the membranes of cells to preserve a high degree of bioavailability for prolonged periods of time while shielding the membrane from Cr‐induced oxidative damage (Sun et al., 2020). According to a recent study, the extracted phytochemicals from ASE had an inhibitory effect on the enzymes xanthine oxidase (XO) and COX 2 in kidney damage and inflammation brought on by cadmium (Osukoya et al., 2021). According to research conducted in vitro, polyphenols, flavonoids, saponins, tannins, alkaloids, and terpenes have anti‐inflammatory properties (Nunes et al., 2020). As a mechanism of inflammatory suppression, antioxidants bind free radicals that increase inflammatory responses and inhibit regulatory enzymes involved in arachidonic acid metabolism, like cyclooxygenases, lipoxygenases, and phospholipase A2. It has been reported that avocado seed flour contains a variety of active chemical components, including flavonoids and saponin, which have anti‐inflammatory activities. However, saponins inhibit cyclooxygenase activity by converting arachidonic acid to endoperoxidase (Tugiyanti et al., 2019). Also, flavonoids have the potential to inhibit enzymes involved in arachidonic acid metabolism, reducing the production of inflammatory mediators originating from this pathway (González Mosquera et al., 2018).

## 5 CONCLUSION

Kidney damage is a result of increased oxidative stress, specifically an insufficient natural antioxidant defense mechanism. It has been proven that ASE's antioxidant activity prevents oxidative kidney injury by minimizing oxidative stress and improving the antioxidant defense system's ability to scavenge free radicals. Avocado seeds are produced in large quantities as a byproduct of industrial processing, and their uncontrolled disposal could harm the environment. These seeds and their constituent parts have anti‐inflammatory and antioxidant qualities and are beneficial as natural food additions and medicinal ingredients. The biochemical, histological, and molecular levels of this study's data indisputably demonstrate that the protective properties of ASE can prevent kidney damage caused by chromium in rabbits. The improvement is due to the antioxidant functions of ASE in treating kidney diseases caused by poisoning. The study focuses solely on identifying the kidney's ameliorative and antioxidant properties. However, to fully understand the signaling pathways and the underlying mechanism of action, further research is warranted.

## AUTHOR CONTRIBUTIONS

**Hanan A. Okail:** Conceptualization (equal); data curation (equal); investigation (equal); resources (equal); writing – original draft (equal). **Sadia Anjum:** Data curation (equal); formal analysis (equal); investigation (equal); project administration (equal); software (equal); visualization (equal); writing – review and editing (equal). **Nahed M. Emam:** Data curation (equal); formal analysis (equal); investigation (equal); methodology (equal); visualization (equal); writing – review and editing (equal). **Rewaida Abdel‐Gaber:** Data curation (equal); formal analysis (equal); funding acquisition (equal); methodology (equal); software (equal); supervision (equal); writing – review and editing (equal). **Mohamed A. Dkhil:** Data curation (equal); formal analysis (equal); investigation (equal); software (equal); validation (equal); visualization (equal); writing – review and editing (equal). **Saeed El‐Ashram:** Data curation (equal); formal analysis (equal); validation (equal). **Mona A. Ibrahim:** Conceptualization (equal); formal analysis (equal); project administration (equal); software (equal); supervision (equal); validation (equal); writing – original draft (equal).

## CONFLICT OF INTEREST STATEMENT

The authors declare no conflicts of interest.

## CONSENT TO PARTICIPATE

All authors agreed to participate in this study.

## CONSENT TO PUBLISH

All authors agreed to publish the data in this study.

## Data Availability

All the datasets generated or analyzed during this study are included in this published article.
